# Quantum phases in circuit QED with a superconducting qubit array

**DOI:** 10.1038/srep04083

**Published:** 2014-02-13

**Authors:** Yuanwei Zhang, Lixian Yu, J. -Q Liang, Gang Chen, Suotang Jia, Franco Nori

**Affiliations:** 1 Institute of Theoretical Physics, Shanxi University, Taiyuan 030006, P. R. China; 2 State Key Laboratory of Quantum Optics and Quantum Optics Devices, Institute of Laser spectroscopy, Shanxi University, Taiyuan 030006, P. R. China; 3 School of Physical Science and Technology, Soochow University, Suzhou, Jiangsu 215006, P. R. China; 4 CEMS, RIKEN, Saitama 351-0198, Japan; 5 Physics Department, The University of Michigan, Ann Arbor, Michigan 48109-1040, USA; 6 Department of Physics, Korea University, Seoul 136-713, Korea

## Abstract

Circuit QED on a chip has become a powerful platform for simulating complex many-body physics. In this report, we realize a Dicke-Ising model with an antiferromagnetic nearest-neighbor spin-spin interaction in circuit QED with a superconducting qubit array. We show that this system exhibits a competition between the collective spin-photon interaction and the antiferromagnetic nearest-neighbor spin-spin interaction, and then predict four quantum phases, including: a paramagnetic normal phase, an antiferromagnetic normal phase, a paramagnetic superradiant phase, and an antiferromagnetic superradiant phase. The antiferromagnetic normal phase and the antiferromagnetic superradiant phase are new phases in many-body quantum optics. In the antiferromagnetic superradiant phase, both the antiferromagnetic and superradiant orders can coexist, and thus the system possesses 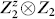 symmetry. Moreover, we find an unconventional photon signature in this phase. In future experiments, these predicted quantum phases could be distinguished by detecting both the mean-photon number and the magnetization.

Circuit quantum electrodynamics (QED) based on superconducting qubits is a fascinating topic in quantum optics and quantum information^1^,^2^,^3^. This artificial spin-1/2 particle can be controlled by tuning the external magnetic flux and gate voltage^4^,^5^,^6^. Moreover, a strong spin-photon coupling has been achieved, which allows to implement quantum operations for long coherence times^7^,^8^. Recently, many important quantum effects in atomic physics and quantum optics have been observed in this artificial spin-photon interaction^9^,^10^. Particularly, experiments realized multiple superconducting qubits interacting with a transmission-line resonator^11^. These experiments allow to explore many-body phenomena via circuit QED^12^,^13^,^14^,^15^,^16^,^17^,^18^,^19^,^20^,^21^. For example, the challenging Dicke quantum phase transition from a normal phase to a superradiant phase, which was predicted more than 30 years ago^22^,^23^,^24^, can be realized by controlling the gate voltage or external magnetic flux^25^,^26^,^27^,^28^ and the no-go theorem arising from the Thomas-Reich-Ruhn sum rule may be overcome^29^,^30^.Moreover, the Jaynes-Cummings lattice model^31^ can also be simulated by an array of transmission-line resonators, each coupled to a single artificial particle^32^,^33^. In addition, by measuring the microwave photon signature, the many-body nonequilibrium dynamics, as well as the known phase diagrams, could be derived^34^,^35^,^36^.

On the other experimental side, superconducting qubits can couple with each other, forming an array with an effective nearest-neighbor spin-spin interaction^37^,^38^. Thus, it is meaningful to explore the many-body physics when a superconducting qubit array couples with a transmission-line resonator because there exists a competition between the collective spin-photon interaction and the nearest-neighbor spin-spin interaction. Recently, sudden switchings, as well as a bistable regime between a ferromagnetic phase and a paramagnetic phase, have been predicted^39^, attributed to this competition.

In this report, we investigate the quantum phases in circuit QED with a superconducting qubit array, which is governed by a Dicke-Ising model with an antiferromagnetic nearest-neighbor spin-spin interaction. By considering the competition between the collective spin-photon interaction and the antiferromagnetic nearest-neighbor spin-spin interaction, we predict four quantum phases, including: a paramagnetic normal phase (PNP), an antiferromagnetic normal phase (ANP), a paramagnetic superradiant phase (PSP), and an antiferromagnetic superradiant phase (ASP). The ANP and the ASP are new phases in many-body quantum optics. In the ASP, both the antiferromagnetic and superradiant orders can coexist, and thus the system possesses 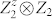 symmetry, i.e., both *U*(1) and translation symmetries are broken simultaneously. Moreover, we find an unconventional photon signature in this phase which could increase from zero to a finite value and then decrease when increasing an effective magnetic field. In future experiments, these predicted quantum phases could be identified by detecting both the mean-photon number and the magnetization.

## Results

### System and Hamiltonian

**Figure 1:**
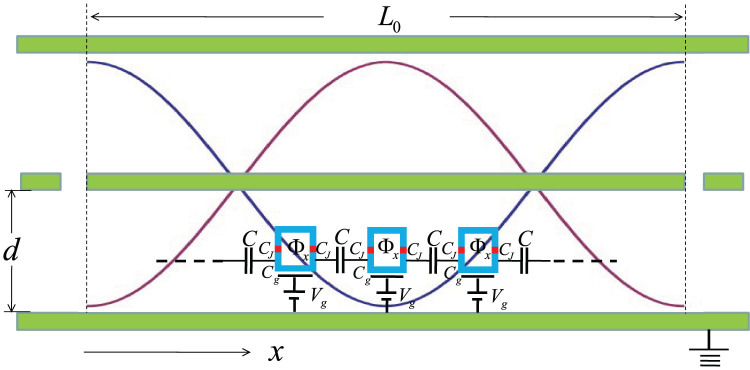
Proposed circuit QED with a superconducting qubit array.

Figure 1 shows our proposed quantum network. Many superconducting qubits connected in a chain couple capacitively to their neighboring qubits and also interact identically with a one-dimensional transmission-line resonator. The corresponding Hamiltonian is given by^4^,^5^,^6^

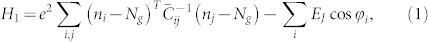
where *n_i_* is the number of Cooper pairs on the *i*th island, 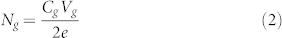
is the dimensionless gate charge with gate capacitance *C_g_* and gate voltage *V_g_*, 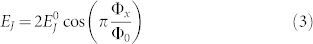
is the tunable Josephson tunneling energy with single-junction Josephson energy 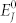, external magnetic flux Φ*_x_*, and magnetic flux quantum Φ_0_, *ϕ_i_* is the phase of the superconducting order parameter of the *i*th island, and 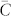 is the capacitance matrix. The element *C_ii_* = *C*_Σ_ is the total capacitance connected to the ith island and *C_ii_*_ ± 1_ = −*C* is the coupling capacitance between two adjacent superconducting qubits. In general, the coupling capacitance *C* is much smaller than the total capacitance *C*_Σ_. As a consequence, the next-nearest-neighbor term of 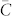 can be neglected safely and the Hamiltonian *H*_1_ is rewritten as 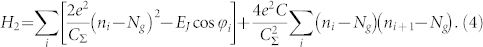
Near the degeneracy point, only a pair of adjacent charge states (*n_i_* = 0 and *n_i_* = 1) on the island are relevant. If we define these charge states as the effective spin basis states 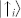 and 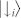, i.e., 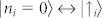 and 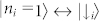, the Hamiltonian *H*_2_ reduces to the form 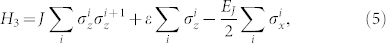
where 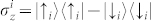 and 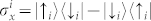 are the Pauli spin operators, 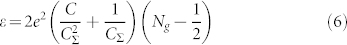
is an effective magnetic field, and 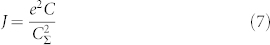
describes the capacitance-induced nearest-neighbor spin-spin interaction.

Now a one-dimensional transmission-line resonator is placed in parallel to the superconducting qubits. All superconducting qubits are situated at the antinode of the magnetic field induced by the oscillating supercurrent in the transmission-line resonator^2^,^3^. Due to the boundary condition at the end of the transmission-line resonator, these superconducting qubits are controlled only by the magnetic component, which shifts the original magnetic flux Φ*_x_* by 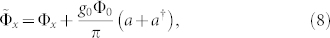
where 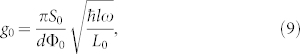
*l* is the inductance per unit length, and *S*_0_ is the enclosed area of the superconducting qubit. In the Lamb-Dicke limit (
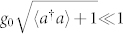
), together with the condition Φ*_x_* = Φ_0_/2, an effective Hamiltonian for Fig. 1 is obtained by 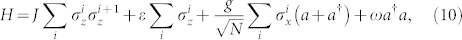
where 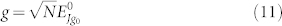
is the collective spin-photon coupling strength. In this Hamiltonian, all parameters can be controlled independently. For example, the effective magnetic field *ε* can be tuned via the gate voltage *V_g_* from the negative to the positive. For simplicity, we address mainly the case *ε* ≥ 0 in the following discussions.

The Hamiltonian (10) is a Dicke-Ising model with an antiferromagnetic nearest-neighbor spin-spin interaction. This Hamiltonian shows clearly that when both *g* and *J* coexist, the collective spin-photon interaction has a competition with the nearest-neighbor spin-spin interaction. As a result, it exhibits exotic phase transitions beyond the previous predictions of the standard Dicke (Ising) model. For example, a first-order superradiant phase transition has been predicted^40^,^41^, when *J* < 0. In this report, we will find rich quantum phases including the PNP, the ANP, the PSP, and the ASP for *J* > 0.

### Quantum phases

For the Hamiltonian (10), the quantum phases can be revealed by calculating the ground-state energy and the order parameters via a mean-field approach^42^. In the classical picture, the spin in the Hamiltonian (10) can be represented as a vector line in the xz plane with the unit vector 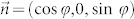. Thus, we can introduce a variational ground-state wave function 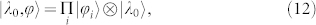
where 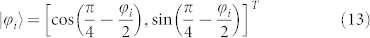
and 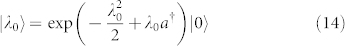
are the spin and boson coherent states, to describe both the antiferromagnetic and superradiant properties.

Since the antiferromagnetic exchange interaction (*J* > 0) leads to a staggered arrangement of all spins in the *z* direction, we should consider two sublattices with *ϕ*_1_ and −*ϕ*_2_, which corresponds to the odd and even sites of spins, respectively, in the ground-state wave function. After a straightforward calculation, the scaled ground-state energy 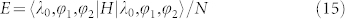
is given by 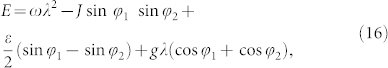
where 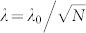 and the parameters (*λ*, *ϕ*_1_ and *ϕ*_2_) are to be determined.

As shown in the Methods section, by minimizing the ground-state energy *E* with respect to the variational parameters (*λ*, *ϕ*_1_, *ϕ*_2_), we obtain three equilibrium equations: 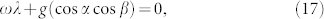


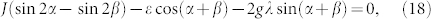


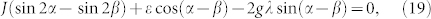
where 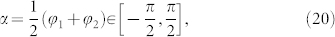


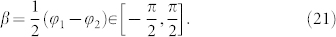
These equilibrium equations, together with the stable conditions (see the Methods section), determine the ground-state energy in Eq. (16) and the order parameters, such as the mean-photon number 〈*a*^†^*a*〉, the magnetization 〈*S_z_*〉 and the staggered magnetization 〈*M_s_*〉, which are given respectively by 
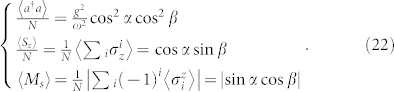
The introduction of the order parameter, the staggered magnetization 〈*M_s_*〉, is to conveniently discuss the antiferromagnetic properties of the Hamiltonian (10). After the ground-state energy, and especially, the order parameters, are obtained, several rich phase diagrams can be obtained.

**Figure 2:**
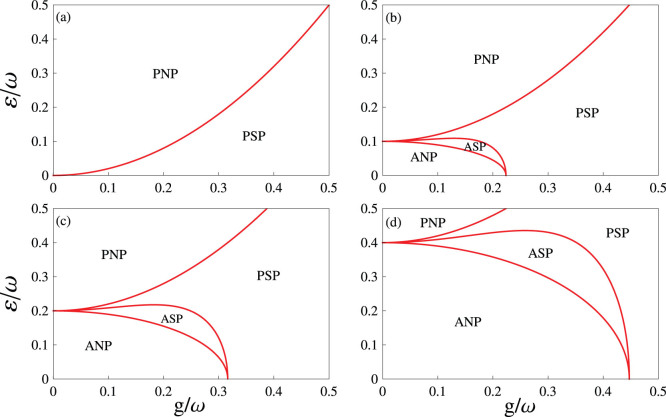
Phase diagrams for different antiferromagnetic spin-spin nearest-neighbor interactions. The plotted parameters are chosen as (a) *J*/*ω* = 0, (b) *J*/*ω* = 0.05, (c) *J*/*ω* = 0.1, and (d) *J*/*ω* = 0.2, respectively. In (b), (c) and (d), the phase boundaries (from top to bottom) are determined respectively by *ε_c_* = 2(*J* + *g*^2^/*ω*), 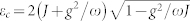, and 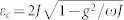.

We first address two known limits. The first is the case when *J* = 0, in which the Hamiltonian (10) reduces to the standard Dicke model^43^

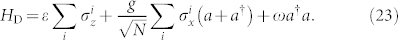
By means of the equilibrium equations (17)–(19) and the stable conditions, we find 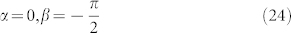
for *g* < *g_c_* and 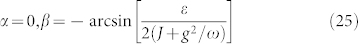
for *g* > *g_c_*, where 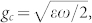 i.e., 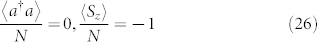
for *g* < *g_c_* and 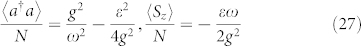
for *g* > *g_c_*. This means that a second-order quantum phase transition from the normal phase (*g* < *g_c_*) to the superradiant phase (*g* > *g_c_*) occurs^44^,^45^, as shown in Fig. 2(a). Moreover, the Dicke model has *U*(1) symmetry in the normal phase. Whereas, in the superradiant phase the system acquires macroscopic collective excitations governed mainly by the collective spin-photon interaction term 
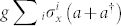
, and thus it has 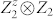 symmetry, where 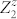 is the global rotation of *π* around the *z* axis^46^ and ^Z^_2_ is the change of sign of the boson coherent state (|*λ*_0_〉 → −|*λ*_0_〉). In experiments, this quantum phase transition has been observed^47^,^48^,^49^ in an optical cavity with a Bose-Einstein condensate by measuring the mean-photon number 〈*a*^†^*a*〉 and the magnetization 〈*S_z_*〉/*N*. Recently, it has been well investigated in many-body circuit QED^25^,^26^,^27^,^28^,^29^,^30^ and spin-orbit-driven Bose-Einstein condensate^50^.

For *g* = 0, the Hamiltonian (10) turns into the Ising model^51^

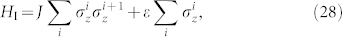
in which a first-order phase transition from the paramagnetic phase to the antiferromagnetic phase at the critical point *J_c_* = *ε*/2 can be recovered. In the paramagnetic phase (*J* < *J_c_*), the ground-state wave function is |… ↓↓↓↓↓↓↓↓ …〉, which implies that the system has translation symmetry and 〈*S_z_*〉/*N* = −1 and 〈*M_s_*〉 = 0. In the antiferromagnetic phase (*J* > *J_c_*), the ground-state wave function becomes |… ↑↓↑↓↑↓↑↓ …〉, in which translation symmetry is broken and 〈*S_z_*〉/*N* = 0 and 〈*M_s_*〉 = 1.

If both *g* and *J* are non-zeros, we find four different regions: (i) *α* = 0, *β* = −*π*/2, (ii) *α* = +*π*/2, *β* = 0, (iii) *α* = 0, *β* ≠ 0, and (iv) *α* ≠ 0, *β* ≠ 0. Specially, the order parameters in these four regions are given respectively by 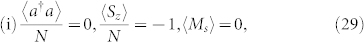


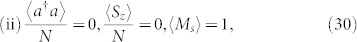


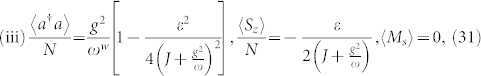


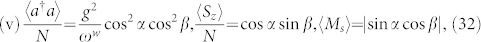
where 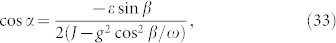


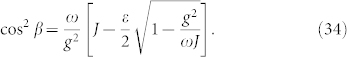
In terms of the different properties of the order parameters, the cases (i)–(iv) are denoted by PNP, ANP, PSP, and ASP, respectively. The ANP (〈*a*^†^*a*〉/*N* = 0, 〈*S_z_*〉/*N* = 0 and 〈*M_s_*〉 = 1) and the ASP (〈*a*^†^*a*〉/*N* ≠ 0, 〈*S_z_〉/N* ≠ −1 and 〈*M_s_*〉 ≠ 1) are new phases in many-body quantum optics. In Fig. 2, we plot phase diagrams for different antiferromagnetic spin-spin nearest-neighbor interactions. This figure shows clearly that these predicted quantum phases can be driven by the collective spin-photon coupling strength *g*, the antiferromagnetic nearest-neighbor spin-spin interaction *J*, and the effective magnetic field *ε*. Especially, the region of the ASP becomes larger when increasing *J*.

**Figure 3:**
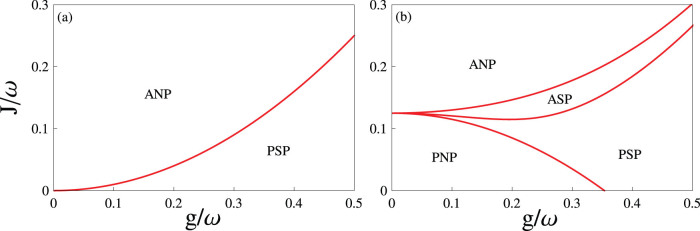
Phase diagrams for different effective magnetic fields. The plotted parameters are chosen as (a) *ε*/*ω* = 0 and (b) *ε*/*ω* = 0.25, respectively. In (a), the phase boundary is determined by *J_c_* = *g*^2^/*ω*. In (b), the phase boundaries (from top to bottom) are given respectively by 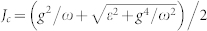, 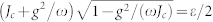, and *J_c_* = *ε*/2 − *g*^2^/*ω*.

In Fig. 3, we plot phase diagrams as functions of the antiferromagnetic nearest-neighbor spin-spin interaction *J* and the collective spin-photon coupling strength *g* for different effective magnetic fields (a) *ε* = 0 and (b) *ε* = *ω*/4. In the absence of *ε*, Eq. (10) reduces to the form 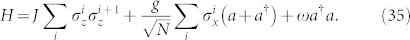
In such a case, only the ANP and the PSP can be found, as shown in Fig. 3(a). When increasing *ε*, four quantum phases are predicted again, as shown in Fig. 3(b). In addition, by means of the ground-state energy, we find that all transitions between these different quantum phases in Figs. 2 and 3 are of second order.

### Symmetry

In order to better understand these predicted quantum phases, it is necessary to discuss the corresponding symmetries. For the PNP and the PSP, the system properties are similar to those of the normal phase and the superradiant phase in the standard Dicke model, i.e., the system displays both *U*(1) and translation symmetries in the PNP, and becomes 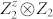 and translation symmetries in the PSP. However, in the ANP, though no photon is excited, the antiferromagnetic order emerges. This implies that in such a case only *U*(1) symmetry can be found. Interestingly, in the ASP the Hamiltonian (10) is governed mainly by the term 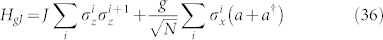
in which there exists a competition between the antiferromagnetic nearest-neighbor spin-spin interaction and the collective spin-photon interaction. As a result, both the antiferromagnetic and superradiant orders coexist and the system possesses 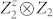 symmetry, i.e., both *U*(1) and translation symmetries are broken simultaneously.

### Possible experimental observation

We first estimate the parameters for experiments. When we choose *C*_Σ_ ~ 600 aF, *C* ~ 20 aF, 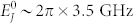, *S*_0_ ~ 1 *μ*m^2^, *d* ~ 10 *μ*m, *L*_0_ ~ 19 mm, *ω* ~ 2*π* × 6.729 GHz, and *N* = 100^11^, the antiferromagnetic nearest-neighbor spin-spin interaction parameter and the collective spin-photon coupling strength are given respectively by *J* ~ 2*π* × 2.2 GHz and *g* ~ 2*π* × 1.5 GHz (*g*_0_ = 0.01 is responsible for the Lamb-Dicke approximation). In addition, the effective magnetic field *ε* can range from 0 to 2*π* × 6.8 GHz by controlling the gate voltage *V_g_*. These parameters ensure that the system should probe the predicted phase transitions. To observe these phase transitions, the relaxation time *T*_1_ and the coherence time *T*_2_ should be much smaller than the lifetime 1/*κ* of the photon, i.e., *T*_1_ > *T*_2_ > 1/*κ* = 23.4 ns, where *κ* is the decay rate of the photon. This restriction can be easy to satisfy in current experimental setups (for example, *T*_1_ = 7.3 *μ*s and *T*_2_ = 500 ns in Ref. 52).

**Figure 4:**
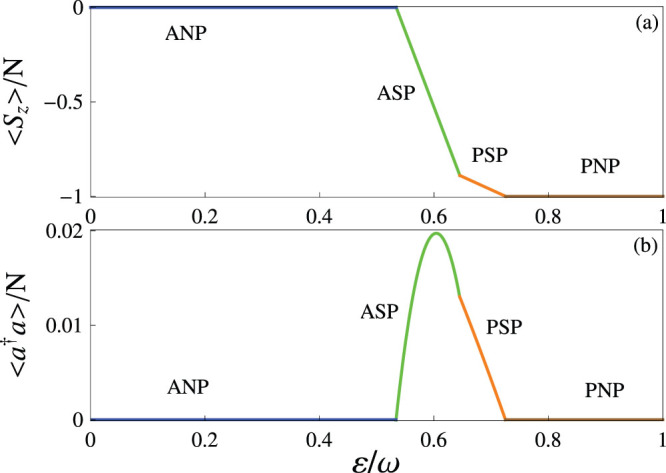
Order parameters versus the effective magnetic field. In (a) and (b), the magnetization 〈*S_z_*〉/*N* and the mean-photon number 〈*a*^†^*a*〉/*N* as functions of the effective magnetic field *ε* are plotted, respectively, when *J*/*ω* = 0.30 and *g*/*ω* = 0.25.

We now illustrate how to identify these different quantum phases. Here we propose to detect four phases by measuring both the mean-photon number 〈*a*^†^*a*〉 and the magnetization 〈*S_z_*〉. For the PNP and the ANP, we can separate these by directly observing the magnetization because 〈*S_z_*〉/*N* = −1 in the PNP and 〈*S_z_〉/N* = 0 in the ANP, as shown in Fig. 4(a). For the PSP and the ASP, both the photon and the spin are collectively excited. Moreover, when increasing the effective magnetic field *ε*, 〈*S_z_*〉/*N* always decreases. This means that it is difficult to distinguish the PSP and the ASP by measuring 〈*S_z_*〉/*N*. Fortunately, we find that in the ASP the mean-photon number has an unconventional behavior that could increase it from zero to a finite value and then decrease, as shown in Fig. 4(b). The relevant physics can be understood as follows. When the effective magnetic field *ε* is applied, it can initially promote the arrangement of all spins from the antiferromagnetic to the paramagnetic terms^53^. For example, in the case of a weak *ε*, the spin arrangement becomes |… ↑↓↑ {↓↓↓} ↑↓↑ …〉 from the antiferromagnetic case |… ↑↓↑↓↑↓↑↓ …〉. This process is helpful for achieving photon-induced collective excitations. Thus, the mean-photon number can increase. However, the rearrangement of spins gives rise to an opposite result of the magnetization, i.e., it decreases when increasing *ε*. For strong *ε*, this effective magnetic field in the *z* axis leads to a large spin imbalance |… ↑↑↓↓↓↓↓↓ …〉 and thus suppresses the spin-photon collective excitations, i.e., both 〈*a*^†^*a*〉/*N* and 〈*S_z_*〉/*N* decrease when increasing *ε*. In terms of the different behaviors of both 〈*a*^†^*a*〉/*N* and 〈*S_z_*〉/*N*, we argue that our predicted quantum phases can be identified.

It should be pointed out that the microwave photon in superconducting circuits is not easy to measure directly^54^ via photon-number detectors, because its energy (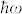) is very small. However, in the dispersive region Δ ≫ *g*, where Δ = *ε* − *ω*, the photon number can be detected by the photon-number-dependent light shift (the Stark plus Lamb shifts) of the atom transition frequency^55^. Unfortunately, to achieve the predicted phase diagrams, the system should work at the quasi-dispersive-strong region Δ/*g* < 4. In such a region, the above approach to detect photons does not work. Recently, it has been proposed^56^,^57^ to detect the photon by irreversible absorption of photons. In these proposals^56^,^57^, the absorbers along the waveguide are built with bistable quantum circuits, and can produce a large voltage pulse when the photon decays into a stable state. This suggests that in future experiments the mean-photon number could be detected, and then our predicted phase diagrams could also be observed.

## Discussion

Let us here address the no-go theorem in quantum optics. This no-go theorem, demonstrated^58^ in 1975, shows that in a typical optical cavity with an ensemble of natural two-level atoms, the phase transition from the normal phase to the superradiant phase is forbidden by the *A*^2^ term, where *A* is the vector potential. Recently, the no-go theorem has been addressed^29^,^30^ in circuit QED, with many superconducting qubits interacting with a quantized voltage (microwave photon). However, in this report, the required microwave photon is generated from the quantization of the magnetic flux. In such a case, no *A*^2^ term can occur, i.e., the no-go theorem is not valid.

We now consider how the decay of the photon and the disorder in fabrication affect the predicted phase diagrams. When considering the decay of the photon, the stationary mean-photon number, which can be derived from 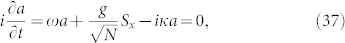
becomes 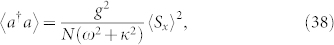
whereas the stationary value of 〈*S_x_*〉 remains unchanged (i.e., it is identical to the case without photon decay). This means that we can use an effective Hamiltonian 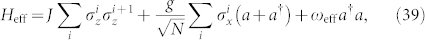
with 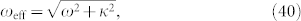
to discuss the phase diagrams^49^ induced by the decay of the photon. Since the decay rate *κ* of the photon (~MHz) is far smaller than the other parameters (~GHz), the decay of the photon has almost no effect on the predicted phase diagrams.

In addition, the imperfections in fabrication result in a weak randomness in the antiferromagnetic nearest-neighbor spin-spin interaction^14^. Moreover, the disorder antiferromagnetic interaction generates a disordered phase, such as a random singlet phase^59^. In this phase, most spins form a singlet pair with nearby spins, and the residual induce weak long-distance pairs. Unfortunately, the disordered phase is only a local correlation, and is thus unstable in the presence of a strong external magnetic field (i.e., for a weak magnetic field, the disordered phase can occur). The phase diagrams predicted here should be observable under a strong magnetic field (See Figs. 2(c) and 2(d)). This means that these predicted quantum phases will not be qualitatively affected by weak disorder.

**Figure 5:**
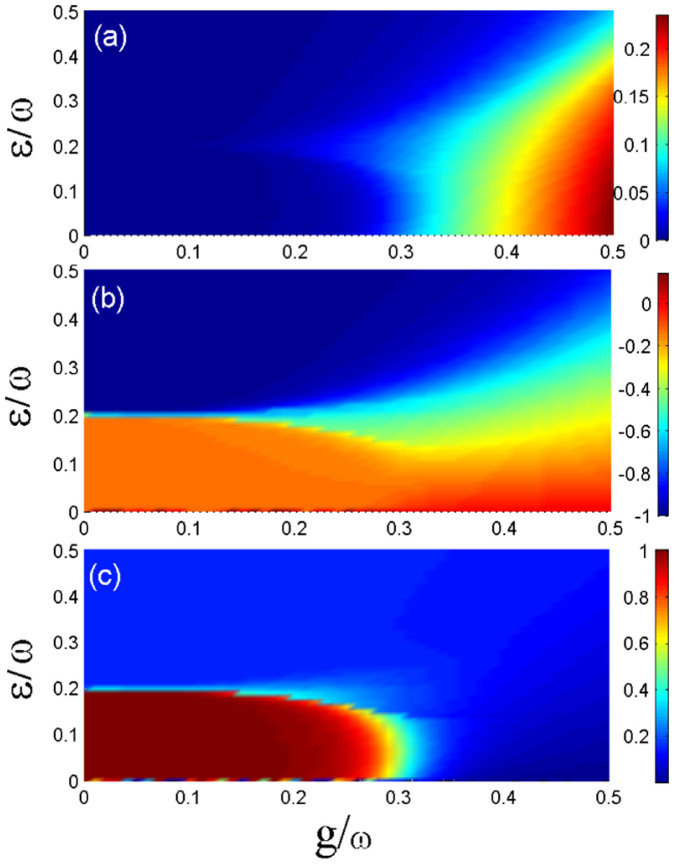
Order parameters via the direct numerical diagonalization method. In (a)–(c), the mean-photon number 〈*a*^†^*a*〉/*N*, the magnetization 〈*S_z_*〉/*N*, and the the staggered magnetization 〈*M_s_*〉 as functions of the collective spin-photon coupling strength g and the effective magnetic field *ε* are plotted, respectively, when *J*/*ω* = 0.1 and *N* = 7.

Mean-field predictions become more accurate for larger number of spins. However, mean-field is often a good starting point, and provides some basic insight in the system. Moreover, for current experimental techniques, the spin number is not sufficiently large, but this should change in the future. For smaller number of spins, we can perform direct numerical diagonalization to discuss the ground-state properties. In Fig. 5, we plot the order parameters 〈*a*^†^*a*〉/*N*, 〈*S_z_*〉/*N*, and 〈*M_s_*〉 as functions of the collective spin-photon coupling strength *g* and the effective magnetic field *ε*. This result shows clearly that for a small number of spins, the predicted quantum phases still exist, but the phase boundaries are affected significantly.

In summary, we have investigated the Dicke-Ising model with an antiferromagnetic nearest-neighbor spin-spin interaction in circuit QED for a superconducting qubit array and predicted four quantum phases, including the PNP, the ANP, the PSP and the ASP, with different symmetries. Moreover, all transitions between these different quantum phases are of second order. We have also found an unconventional photon signature in the ASP, where both the antiferromagnetic and superradiant orders coexist. We believe that this system allows to explore exotic many-body physics in quantum optics and condensed-matter physics because it has an interesting competition between the collective spin-photon interaction and the nearest-neighbor spin-spin interaction.

## Methods

### Three equilibrium equations and the corresponding stable conditions

Here we present detailed calculations on how to derive the three equilibrium equations (17)–(19) and the corresponding stable conditions. After minimizing the ground-state energy *E* in Eq. (16) with respect to the variational parameters (*λ*, *ϕ*_1_ and *ϕ*_2_), we obtain three equations: 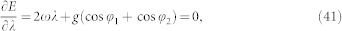


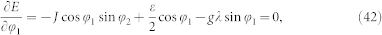


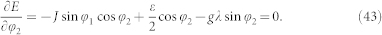
After defining *α* = (*ϕ*_1_ + *ϕ*_2_)/2 **∈** [−*π*/2, *π*/2] and *β* = (*ϕ*_1_ − *ϕ*_2_)/2 **∈** [−*π*/2, *π*/2], the three equilibrium equations (17)–(19) are easily derived.

In addition, by means of three parameters (*λ*, *α* and *β*), the ground-state stability is determined by the 3 × 3 Hessian matrix 
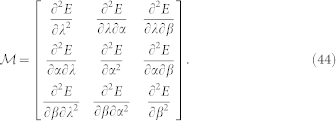
If 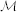 is positive definite (i.e., all eigenvalues *h_i_* of 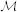 are positive), the system is located at the stable phases. If 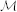 is negative definite (i.e., all eigenvalues *h_i_* of 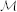 are negative), the system is unstable.

## Author Contributions

G.C., S.J. and F.N. conceived the idea. Y.Z., L.Y. and J.-Q.L. performed the calculation. G.C., S.J. and F.N. wrote the manuscript.
